# Adenovirus Encoding Tumor Necrosis Factor Alpha and Interleukin 2 Induces a Tertiary Lymphoid Structure Signature in Immune Checkpoint Inhibitor Refractory Head and Neck Cancer

**DOI:** 10.3389/fimmu.2022.794251

**Published:** 2022-03-07

**Authors:** James H. A. Clubb, Tatiana V. Kudling, Camilla Heiniö, Saru Basnet, Santeri Pakola, Víctor Cervera Carrascón, João Manuel Santos, Dafne C. A. Quixabeira, Riikka Havunen, Suvi Sorsa, Vincent Zheng, Tuula Salo, Leif Bäck, Katri Aro, Sanni Tulokas, Venla Loimu, Akseli Hemminki

**Affiliations:** ^1^ Cancer Gene Therapy Group, Faculty of Medicine, University of Helsinki, Helsinki, Finland; ^2^ TILT Biotherapeutics Ltd, Helsinki, Finland; ^3^ Translational Immunology Research Program (TRIMM), Research Program Unit (RPU), University of Helsinki, Helsinki, Finland; ^4^ Department of Oral and Maxillofacial Diseases, Clinicum, University of Helsinki, Helsinki, Finland; ^5^ Cancer and Translational Medicine Research Unit, University of Oulu, Oulu, Finland; ^6^ Oulu University Central Hospital, Oulu, Finland; ^7^ Department of Oncology, Comprehensive Cancer Centre, Helsinki University Hospital and University of Helsinki, Helsinki, Finland; ^8^ Department of Otorhinolaryngology – Head and Neck Surgery, Helsinki Head and Neck Center, Helsinki University Hospital and University of Helsinki, Helsinki, Finland

**Keywords:** adenovirus, immune checkpoint inhibitor, head and neck cancer, immunotherapy, tertiary lymphoid neogenesis, TNFa, IL2, oncolytic virotherapy

## Abstract

Immune checkpoint inhibitors (ICI) have provided significant improvement in clinical outcomes for some patients with solid tumors. However, for patients with head and neck cancer, the response rate to ICI monotherapy remains low, leading to the exploration of combinatorial treatment strategies. In this preclinical study, we use an oncolytic adenovirus (Ad5/3) encoding hTNFα and hIL-2 and non-replicate adenoviruses (Ad5) encoding mTNFα and mIL-2 with ICI to achieve superior tumor growth control and improved survival outcomes. The *in vitro* effect of Ad5/3-E2F-D24-hTNFa-IRES-hIL-2 was characterized through analyses of virus replication, transgene expression and lytic activity using head and neck cancer patient derived cell lines. Mouse models of ICI naïve and refractory oral cavity squamous cell carcinoma were established to evaluate the local and systemic anti-tumor immune response upon ICI treatment with or without the non-replicative adenovirus encoding mTNFα and mIL-2. We delineated the mechanism of action by measuring the metabolic activity and effector function of CD3^+^ tumor infiltrating lymphocytes (TIL) and transcriptomic profile of the CD45^+^ tumor immune compartment. Ad5/3-E2F-D24-hTNFa-IRES-hIL-2 demonstrated robust replicative capability *in vitro* across all head and neck cell lines screened through potent lytic activity, E1a and transgene expression. *In vivo*, in both ICI naïve and refractory models, we observed improvement to tumor growth control and long-term survival when combining anti-PD-1 or anti-PD-L1 with the non-replicative adenovirus encoding mTNFα and mIL-2 compared to monotherapies. This observation was verified by striking CD3^+^ TIL derived mGranzyme b and interferon gamma production complemented by increased T cell bioenergetics. Notably, interrogation of the tumor immune transcriptome revealed the upregulation of a gene signature distinctive of tertiary lymphoid structure formation upon treatment of murine anti-PD-L1 refractory tumors with non-replicative adenovirus encoding mTNFα and mIL-2. In addition, we detected an increase in anti-tumor antibody production and expansion of the memory T cell compartment in the secondary lymphoid organs. In summary, a non-replicative adenovirus encoding mTNFα and mIL-2 potentiates ICI therapy, demonstrated by improved tumor growth control and survival in head and neck tumor-bearing mice. Moreover, the data reveals a potential approach for inducing tertiary lymphoid structure formation. Altogether our results support the clinical potential of combining this adenovirotherapy with anti-PD-1 or anti-PD-L1.

## Introduction

Head and neck cancer is the sixth most common cancer worldwide and is anticipated to increase 30% by 2030 to 1.08 million new cases annually (1, 2). The majority of cases (90%) are head and neck squamous cell carcinomas (HNSCC) located in the upper aerodigestive tract (3). Existing treatment strategies for HNSCC rely on surgical resection, sometimes in combination with radiotherapy and/or chemotherapy, or radical chemoradiation. However, the majority of patients with advanced stage disease develop recurrence or distant metastases (4–6). Consequently, the 5-year overall survival remains modest (~60%), while patients with unresectable, advanced and metastatic disease, have a significantly poorer prognosis with an expected survival of less than 12 months (7, 8). Therefore, there is an urgent need to develop additional treatment strategies capable of improved therapeutic efficacy against both HPV positive and negative HNSCC.

Recent advances in immunotherapy, such as immune checkpoint inhibitors (ICI), have provided clinical benefits for a subpopulation of HNSCC patients, in the form of survival (9, 10). However, only approximately 20% of HNSCC patients receive measurable benefits from treatment with programmed death 1 (PD-1) or programmed death ligand 1 (PD-L1) blocking antibodies (11). Moreover, patients initially responding to ICI may develop disease progression, becoming refractory (12). ICI resistance has been categorized into innate (primary) and acquired (secondary) resistance (13). Mechanistically resistance derives from genetic and epigenetic alternations which can prevent T cell recruitment, lead to inadequate metabolic and effector immune cell functions and ultimately shape the tumor microenvironment towards immunosuppression (13). As a result, multiple studies aiming to increase the potency and duration of response to ICIs through combinatorial approaches are now under investigation.

Oncolytic viruses (OVs) are a class of immunotherapeutic agents, capable of inducing a potent immune response, through selective lysis of tumor cells, release of tumor-associated antigens and facilitation of epitope spreading (14). OVs can be further modified to contain immunostimulatory transgenes, designed to further amplify the antitumor immune response. Adenoviruses make up one family of well-characterized OVs with these capabilities, yet also have distinct inherencies, including an ability to prime CD4^+^ T cells with mGranzyme-mediated cytotoxic function and PD-1 sensitivity (14, 15).

We have previously developed a novel chimeric oncolytic adenovirus (Ad5/3-E2F-D24-hTNFa-IRES-hIL-2; also referred to as TILT-123) encoding tumor necrosis factor alpha (TNFα) and interleukin-2 (IL-2) (16, 17). This virus has shown promising safety and survival in pre-clinical studies. Additionally, the virus selectively replicates in cancer cells, resulting in the release of danger-associated-molecular-patterns (DAMPs) and pathogen-associated-molecular-patterns (PAMPs) and promotes the influx and propagation of a potent anti-tumor immune cells in the tumor (18–20). Consequently, phase 1 clinical trials using Ad5/3-E2F-D24-hTNFa-IRES-hIL-2 as a monotherapy and in combination with tumor infiltrating lymphocyte (TIL) therapy are currently ongoing in solid tumors and in metastatic melanoma patients (NCT04695327, NCT04217473, respectively).

We have previously reported that combining adenovirus-mediated delivery of mTNFα and mIL-2 with anti-PD-1 or anti-PD-L1 is beneficial, in models of ICI naïve and resistant murine B16.OVA melanoma (21, 22). Here we investigated the effectiveness of adenovirus mediated delivery of IL-2 and TNFα for treating HNSCC. For this purpose, we employed a highly immunogenic (MOC1) (ICI sensitive) and poorly immunogenic (MOC2) (primary ICI resistant) syngeneic model of treatment naïve oral cavity cancer, in which we observed superior tumor responses and survival when combining ICI (anti-PD-L1 or anti-PD-1) with Ad5-CMV-mTNFα/mIL-2 (a murine version of TILT-123).

Since acquired ICI resistance is the situation with the most unmet clinical need, we developed a mouse model allowing testing of our hypothesis in the refractory setting. Analysis of TILs revealed greater activation and bioenergic status, accompanied by increased Interferon gamma (IFNγ) and mGranzyme b, following combination therapy. Notably, differential gene expression analysis of ICI resistant MOC1 tumors treated with anti-PD-L1 and Ad5-CMV-mTNFα/mIL-2 revealed a tertiary lymphoid structure (TLS) hallmark gene signature associated with increased anti-tumor antibody production and Natural Killer cell (NK)-mediated cytotoxicity. These findings support the rationale for clinical investigation of the combination of ICI therapies with Ad5/3-E2F-D24-hTNFa-IRES-hIL-2 for treating HNSCC patients.

## Methods

### Cell Lines and Viruses

The UT-SCC cell lines were established from HNSCC tumors at the Department of Otorhinolaryngology-Head and Neck Surgery, Turku University Hospital (Turku, Finland) by Prof. Reidar Grènman (23). HSC-3 is from the Japanese Health Science Research Resources Bank, Osaka, Japan. All these cells were cultured in Dulbecco’s Modified Eagle Medium (DMEM) supplemented with 2 mM L-glutamine, 10% fetal bovine serum, non-essential amino acids solution, 1% penicillin, and 1% streptomycin. Their clinical information can be found in **Table 1**. MOC1 and MOC2 were purchased from ATCC and cultured according to the recommended conditions. The construction of Ad5/3-E2F-D24-hTNFa-IRES-hIL-2, Ad5/3-E2F-D24, Ad5-CMV-mIL-2, Ad5-CMV-mTNFα and Ad5-Luc is described elsewhere (16, 17).

**Table 1:** Head and neck cell line characteristics.

Cell line name	Age^1^	Diagnosis	Pre-Treatment^2^	HPV+/- ^3^	Resection	Location
HSC-3	64	Tongue squamous cell carcinoma	?	–	Metastasis	Cervical lymph node
UT-SCC-8	42	Laryngeal squamous cell carcinoma (T2N0M0)	None	–	Primary	Epiglottis
UT-SCC-9	81	Laryngeal squamous cell carcinoma (T2N0M0)	radiosensitive	–	Primary	Larynx, glottis
UT-SCC-10	62	Oral cavity; tongue	?	–	Primary	Tongue
UT-SCC-24A	41	Tongue squamous cell carcinoma (T2N0M0)	?	–	Primary	Tongue
UT-SCC-24B	41		?	–	Metastatic	Cervical lymph node
UT-SCC-42A	43	Laryngeal squamous cell carcinoma (T4N3M0)	?	–	Primary	Larynx; supraglottis
UT-SCC-42B	43		?	–	Metastatic	Cervical lymph node

^1^Age = length of time patient was alive at time of surgery.

^2^Pre-Treatment (?) = information not confirmed.

^3^HPV status of all cell lines was determined by PCR. (+) = positive/(-) = negative.

Note. Information was derived from previously published data. More information about the UT-SCC cell lines can be found in this publication (24).

### Cell Viability Assay

For human HNSCC cell line analysis, cells were seeded at 1x10^4^ cells/well, were left to rest for 24 hours in 37°C and infected with 1, 10, 100 and 1000 VP/cell of Ad5/3-E2F-D24-hTNFa-IRES-hIL-2 or Ad5/3-E2F-D24. Then, the cell viability of the HNSCC cell lines was determined by MTS on day 2, 6, 10. Wells were incubated for 2 hours with 40 µl of CellTiter 96 AQueous One Solution Proliferation Assay reagent (Promega, Wisconsin, USA). Absorbance was read at 492 nm using a Fluostar OPTIMA analyzer (BMG Labtech, Offenburg, Germany). Each time data-point was normalized to the vehicle control group.

### Cell Proliferation Assay

Ten thousand cells were seeded on E-Plate L8 disposable plates (ACEA Biosciences, Agilent technologies) for 24 hours, then infected with Ad5-CMV-mIL-2, Ad5-CMV-mTNFα or Ad5-Luc at 10,000, 1000, 100 and 10VP/cell. Kinetic analysis of the infected cancer cells was then performed and cancer cell line growth inhibition was measured as relative cell impedence using xCELLigence real-time cell analyzer. Data was normalized to the 24 hour point.

### Murine Tumor Models

4–6 weeks old female C57BL/6JOlaHsd (immunocompetent) mice were purchased from Envigo (Indianapolis, IN, USA). For the syngeneic mouse model, B6 mice were subcutaneously (right flank) injected with either 2x10^6^ MOC1 cells or 1x10^5^ MOC2 cells in 50μl PBS. Established tumors (stratified randomization) were treated either with intratumoral injection of PBS, 1 × 10^9^ VP of unarmed control virus (Ad5-Luc), 1:1 mixture of non-replicative Ad5-CMV-mTNFα and Ad5-CMV-mIL-2 virus (total 1 × 10^9^ VP) and/or intraperitoneal injection of anti-PD-1 Ab (clone RMP1-14, BioXcell) or anti-PD-L1 Ab (clone 10 F.9G2, BioXcell) (100 µg/injection). Intended number of animals per group was n= 10 but engraftment rate was not 100% (n=8 PBS versus, n=9 antiPD-1/PD-L1, n=8 Ad5-Luc, n=9 Ad5-CMV-mTNFα/mIL-2, n=9 combination). Tumor dimensions were measured with digital calipers and the volumes were calculated as (length × width^2^)/2. When maximum allowed tumor diameter of 18 mm was reached, animals were immediately euthanized and samples for further analysis were collected. Animals with open wounds (ulcers at the injection site) were euthanized as per animal permit and excluded from the study. Total animals for treatment naïve MOC1 n= 60, MOC2 n=40, refractory MOC1 n=120.

### Histopathology

MOC1 tumor fragments were fixed in 10% formalin for 24 hours and preserved in 70% ethanol until further paraffin embedding. Immunodetection was carried out using the Vectastain ABC-HRP (avidin/biotin-peroxidase) kit (Vector Laboratories, Burlingham, CA; ref. PK-4000) according to manufacturer’s instructions. Antigen retrieval for all antibodies was done in citrate buffer pH 6.0, 20 min at 99 °CC. Hematoxylin and eosin (HE), CD3 Rabbit polyclonal (Agilent Dako Code A0452; dilution 1:400), Ki67 Rabbit monoclonal (Clone SP6, Thermo Fisher Scientific Cat number RM-9106-S1; dilution 1:200), CD45r Rat monoclonal (Clone RA3-6B2, BIO-RAD Product code MCA1258G; dilution 1:600), CD19 Rabbit monoclonal (EPR23174-145, Abcam, ab245235; 1:300 dilution), staining were performed in 4–5 µm thickness sections cut from the paraffin blocks. The secondary for Ki67 and CD3 was biotinylated Goat Anti-Rabbit Ab (Vector Laboratories; Cat no BA-1000-1.5) and for CD45R biotinylated mouse adsorbed Goat Anti-Rat Ab (Vector Laboratories; Cat no BA-9401-.5). Images were generated using 3DHISTECH Pannoramic 250 FLASH II digital slide scanner at Genome Biology Unit supported by HiLIFE and the Faculty of Medicine, University of Helsinki, and Biocenter Finland. Quantification of CD19^+^ was performed using computer assisted image analysis using ImageJ Fiji v1.53c (Wayne Rasband National Institutes of Health, USA). Percentage positivity was determined by thresholding, removing artifacts and gating around the tumor section.

### Cytokine Analysis

Treated MOC1 tumors were fragmented and snap-frozen on dry ice and stored at -80°C until further analysis. Tumor fragments were thawed and processed as previously described by our group (25). Samples were stained with Cytometric Bead Array Mouse Th1/Th2/Th17 Cytokine kit (560485, BD) and analyzed on BD Accuri C6 Cytometer (BD, Franklin Lakes, NJ, USA) with FCAP Array Software (BD, Franklin Lakes, NJ, USA) according to the manufacturer’s instructions. Similarly, Human IL-2 and TNFα Flex Sets (558270, 558273, BD) were used for analysis of human IL-2 and TNFα produced by Ad5/3-E2F-D24-hTNFa-IRES-hIL-2 in the human HNSCC cell lines. Cytokine concentration was normalized to the total protein concentration of the culture supernatant, as measured by Qubit™ Protein Assay Kit (Invitrogen™).

### Quantitative Real-Time PCR of E1a d24

Human HNSCC patient derived cell lines were cultured as described above. Cells were infected with 100 VP/cell of Ad5/3-E2F-D24-hTNFa-IRES-hIL-2 and collected at day 1, 2 and 3. DNA was isolated from cells using QIAmp DNA Mini Kit (51306, Qiagen) and were incubated 10min at 95°C; 50 cycles of 10 sec at 95°C; 50 cycles of 30 sec 62°C; 50 cycles of 20 sec at 72°C and 10min at 40°C with primers targeting the d24 deletion of the E1A region; forward primer (5′-TCCG GTTTCTATGCCAAACCT-3′), reverse primer (5′-TCCT CCGGTGATAATGACAAGA-3′) and probe (5′FAMTGATCGATCCACCCAGTGA-3′MGBNFQ) (metaBion Oligomer, Germany). Plates were subsequently measured using LightCycler 480 II System (Roche). Quantification of E1a was calculated according to a standard curve generated using known concentrations of a plasmid coding for our virus.

### CD45^+^ T Cell Isolation and Processing From Tumor and Lymphoid Tissue

For analysis of intratumoral T cells, tumors were mechanically disrupted using gentleMACs C tubes (Milltenyi Biotech) followed by enzymatic digestion using collagenase type IV (1mg/mL), Hyaluronidase (2U/mL) and DNase I (10U/mL) in RPMI with 10% FBS for 1 hour. After double filtering of disrupted tumor tissue, T cells were isolated using Pan T Cell Isolation Kit II or CD45 Microbeads, mouse (130-095-130, 130-052-301 Miltenyi Biotec). Spleens and lymph nodes were pressed through 70μm filters and filtered flow-through was then used fresh for downstream analysis.

### Enzyme-Linked Immunospot (ELISpot) Assay and IL-21 ELISA

Mouse IFN-γ/GrB dual-color Elispot kit was performed according to the manufacturer’s instructions (ELD5819, R&D Systems, Minneapolis, MN, USA). For analysis of intratumoral T cells and splenocytes, 5x10^4^ cells freshly isolated CD3^+^ cells or whole splenocytes were plated per well for 24 hours. Cells were cultured in RPMI 1640 supplemented with 20% FBS, 1% L-glutamin, 1% Pen/strep, 15 mM HEPES, 1 mM Na-pyruvate, 50 um b-Mercaptoethanol (Sigma-Aldrich, Missouri, USA). IL-21 from MOC1 tumor digests were analysed using the LEGEND MAX™ Mouse IL-21 ELISA Kit (446107).

### Flow Cytometry

For analysis of tumors and lymphoid organs derived from the MOC1 syngeneic model, antibodies specific for mouse CD3 (560590; 17A2), CD4 (740024; H129.19), CD8a (55795; 53-6.7), CD44 (560780; IM7), CD62L (562404; MEL-14), CD69 (552879; H1.2F3), CD25 (562694; PC61), Foxp3 (560403; MF23), T-bet (561264; 4B10), and CXCR5 (560617; 2G8), were purchased from BD Biosciences, California, USA. Intracellular staining of transcription factors was performed using Fixation/Permeabilization solution kit (554714) or Mouse Foxp3 Buffer set (560409) from BD Biosciences. All samples were stained after Fc blocking using 5ul/test of purified rat anti–mouse CD16/32 antibody (BD Biosciences). Samples were acquired using FACS Aria II cell sorter (BD Biosciences, California, USA) and data analysis was performed using Flowjo^®^ software v10 (Flowjo LLC, BD Biosciences, California, USA). MFI is presented as the median value of the samples.

### Bulk RNA-Sequencing of CD45^+/-^ Tumor Fractions

RNA from CD45^+/-^ MOC1 tumor cells was purified using RNeasy kit according to the manufacturer’s instructions (74106, Qiagen, Hilden, Germany). Concentrations were adjusted following measurement using Qubit 4 Flourometer and consolidated with Agilent 4200 Tapestation. Sequencing and analysis was performed by GENEWIZ (Germany) using PolyA selection and 20-30 million reads per sample. Sequence reads were trimmed to remove possible adapter sequences and nucleotides with poor quality using Trimmomatic v.0.36. The trimmed reads were mapped to the Mus musculus GRCm38 reference genome available on ENSEMBL using the STAR aligner v.2.5.2b. Unique gene hit counts were calculated by using featureCounts from the Subread package v.1.5.2. The hit counts were summarized and reported using the gene_id feature in the annotation file. Only unique reads that fell within exon regions were counted. If a strand-specific library preparation was performed, the reads were strand-specifically counted. Using DESeq2, a comparison of gene expression between groups was performed. Distribution of read counts in libraries were examined before and after normalization. The original read counts were normalized to adjust for various factors such as variations of sequencing yield between samples. These normalized read counts were used to accurately determine differentially expressed genes. The Wald test was used to generate p-values and log2 fold changes. Genes with an adjusted p-value < 0.05 and absolute log2 fold change > 1 were called as differentially expressed genes for each comparison.

### Metabolic Assays

Filtered murine tumor digests (1x10^6^ total cells) were cultured with 20 μM 2-NBDG in 5% FBS-containing media for 45 min at 37°C. Cells were further stained for surface markers of interest. T cell metabolic function was measured using Seahorse XFe96 Analyzer and either XFp Real-Time ATP Rate Assay Kit or Cell Mito Stress Test Kit (Agilent Technologies, California, USA). T cells (1.5x10^5^) were seeded on Cell-Tak coated culture plates in assay media consisting of unbuffered RPMI supplemented with 1% BSA and 25mM glucose, 1 μM pyruvate, and 2 mM glutamine. Basal extracellular acidification and oxygen consumption rates were collected for 30 min. Cells were stimulated with oligomycin (2 μM) and rotenone/antimycin A (100 μM) to obtain maximal respiration.

### Anti-MOC1 Associated IgG Quantification

For quantification of anti-MOC1 IgG, MOC1 cells (1x10^4^ cells/well) were seeded in 96 well plates overnight then fixed in 4% PFA for 20mins. Tumor digests supernatants from treatment groups were serially diluted and incubated with fixed cells overnight at 4°C. The following day, IgG was quantified using the In-Cell ELISA Kit, Colorimetric (662200, Invitrogen) according to the manufacturer’s instructions.

### NK Cell Cytotoxicity Assay

For the NK cytoxicity assay, MOC1 (1x10^4^ cells/well) were seeded in 96 well plates for 24 hours as target cells. The next day the cells were fluorescently labelled with 10uM CFSE using CellTrace™ CFSE Cell Proliferation Kit (C34554, Invitrogen) then incubated for addition 1hr. Target cells were then incubated with serially diluted tumor supernatants and/or NK cells (1:1 E/T ratio) isolated from C57BL/6 spleens using NK Cell Isolation Kit, mouse (130-115-818, Miltenyi Biotec) overnight. After 24 hours the plate was analysed using the Alexa Flour 488 setting of Hidex Sense plate reader and the treatment group values were normalized to MOC1 cells and NK cells without supernatants. The assays were performed in at least three technical replicates with NK cells pooled from three mice.

### Statistical Analysis

Statistical analysis was performed with Prism 8 (GraphPad Software) and heatmaps were generated using RStudio. Unpaired Student’s t test was used to compare 2 groups and 1-way ANOVA with Tukey’s *post hoc* test was used to compare 3 or more groups. Normality test was used to determine distribution of data for parametric tests vs non-parametric tests. Tumor growth curves were compared using mixed-model analysis. Survival curves were generated using the Kaplan-Meyer method and the differences of 2 curves were compared using the log-rank test. *P* values < 0.05 were considered significant. Correlations between variables were investigated using Spearman non-parametric correlation.

## Results

### Ad5/3-E2F-D24-hTNFa-IRES-hIL-2 Efficiently Replicates and Lyses Primary and Metastatic Head and Neck Squamous Cell Carcinoma Cell Lines

**Figure 1:**
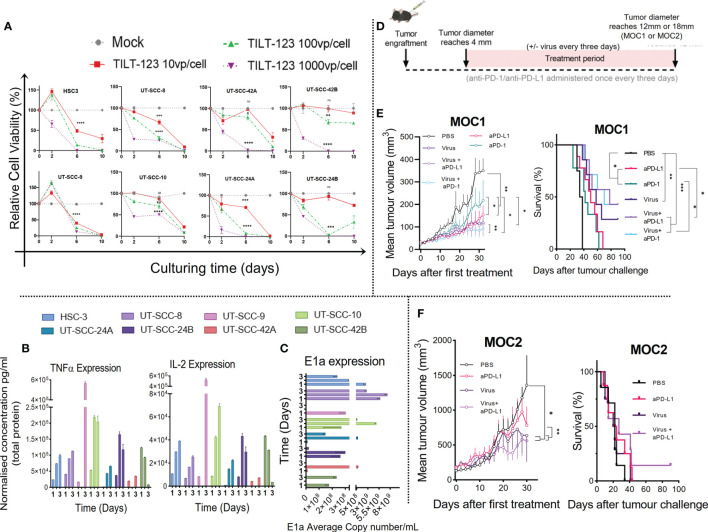
Ad5/3-E2F-D24-hTNFa-IRES-hIL-2 replicates in all HNSCC cell lines screened and the antitumor response in ICI treatment naïve murine model is improved when combining ICI with non-replicative Ad5-CMV-mTNFα/mIL-2. **(A)** Lytic activity measured by MTS of oncolytic adenovirus (Ad5/3-E2F-D24-hTNFa-IRES-hIL-2) in HNSCC patient derived cell lines cultured over 10 days with either 10, 100 or 1000VP/cell. **(B)** Quantification of IL-2 and TNFα by cytometric bead array of cell culture supernatants normalized to total protein measured by Qubit flourometer. **(C)** viral replication (E1a qPCR) measured from isolated DNA from infected HNSCC cell lines infected with 100VP/cell after 24hours, 48hours and 72hours. **(D)** Experiment design. C57BL/6J mice (n = 7-10 per group) were subcutaneously injected with either e MOC1 or f MOC2 cells into the right flank. When tumors reached 4-5mm then 1 × 10^9^ VPs of non-replicating Ad5-CMV-mTNFα/mIL-2 or PBS were injected intratumorally with or without intraperitoneal injection of (100ug) murine anti-PD-1 or anti-PD-L1. Treatment frequency as indicated. **(E, F)** presented as mean tumor volume after 30 days and overall survival after 100 days treatment. *In vitro* data sets were assayed in triplicates and evaluated for statistical significance by non-parametric unpaired t tests. *In vivo* tumor growth curves and survival curves were evaluated for statistical significance by two-way mixed model ANOVA and Mantel–Cox log-rank test respectively. All data sets are presented as means ± SEM and significance represented as *p < 0.05, **p < 0.01, ***p < 0.001, and ****p < 0.0001, ns, not significant.

The lytic capacity of Ad5/3-E2F-D24 (control without transgenes), (**Supplementary Figure S1A**) and Ad5/3-E2F-D24-hTNFa-IRES-hIL-2 (**Figure 1A**) in 8 HNSCC patient-derived cell lines with diverse clinical characteristics (**Table 1**), were determined *in vitro*. This revealed that both Ad5/3-E2F-D24 and Ad5/3-E2F-D24-hTNFa-IRES-hIL-2 significantly (p<0.05 and p<0.01 respectively) decreased the viability of all HNSCC cell lines to <50% by day 6 and all to 0% by day 10 at 1000VP/cell and in a dose dependent manner (relative to vehicle control). Ad5/3-E2F-D24 and Ad5/3-E2F-D24-hTNFa-IRES-hIL-2 showed similar cell killing efficacy, however HSC-3, UT-SCC-10 and UT-SCC-24B were more effectively killed by the armed virus by day 6 at the three tested doses (**Figure 1A**). A summary showing the average cytotoxicity of Ad5/3-E2F-D24 and Ad5/3-E2F-D24-hTNFa-IRES-hIL-2 in all 8 cell lines can be seen in **Supplementary Figure 1D**. The specificity of Ad5/3-E2F-D24 and Ad5/3-E2F-D24-hTNFa-IRES-hIL-2 replication and transgene expression has recently been reported (26).

Next, in an independent experiment, we analyzed the supernatants from the same cell lines infected with 100 VP/cell collected at day 1, 2 and 3 after infection, for virally produced human IL-2 and TNFα (**Figure 1B**). In parallel, we isolated the DNA for measurement of replication by quantifying viral early gene (E1a) by qPCR (**Figure 1C**). We observed transgene expression and virus replication across all 8 cell lines, with cytokine supernatant concentration reaching maximum at either day 2 or day 3. Cytokine concentrations for TNFα and IL-2 at day 3 ranged from 8.08 ng/mL to 563.71 ng/mL and 3.57 ng/mL to 525.49 ng/mL respectively, and a higher concentration of TNFα was generated in comparison to IL-2 at each time point. This is likely because the TNFα transgene is placed before the IL-2 in the virus construct. Notably, UT-SCC-9 and UT-SCC10 generated the highest and UT-SCC-24A and UT-SCC-42B the lowest cumulative cytokine concentration and E1a copy number over the three time points (**Figures 1B, C**). Interestingly, high adenovirus induced toxicity with low E1a and cytokine expression was observed in UT-SCC-24a. This could be caused by high sensitivity of the cell line to other virus-induced cell death mechanisms such as necroptosis, pyroptosis and autophagy (27). No significant correlation between E1a expression and cytokine expression was observed collectively across screened cell lines (**Supplementary Figures S3B, C**). Collectively, these results show that Ad5/3-E2F-D24-hTNFa-IRES-hIL-2 is capable of replicating in and lysing several types of human head and neck cancer, with subsequent release of TNFα and IL-2, despite histology or tumor location.

### MOC Tumours Respond to Immune Checkpoint Inhibition and Non-Replicative Ad5-CMV-mTNFα/mIL-2

To evaluate our virotherapy’s potential to improve the therapeutic response to ICI in HNSCC *in vivo*, we performed tumor-growth and overall survival measurements in two murine models (MOC1 and MOC2) of oral cavity cancer (using the non-replicative adenovirus encoding mTNFα and mIL-2)(**Figure 1D**). We hypothesized that combining anti-PD-1/PD-L1 with Ad5-CMV-mTNFα/mIL-2 treatment would improve tumor growth control and animal survival, when compared to monotherapies. As shown in **Figure 1E** MOC1 tumor-bearing mice significantly benefited from anti-PD-1 and anti-PD-L1 monoclonal antibody (mAb) monotherapy. The anti-PD-1 and anti-PD-L1 treated mice had significantly smaller tumors compared to mock (p<0.01 and p<0.01 respectively) by day 30. Ad5-CMV-mTNFα/mIL-2 monotherapy provided better response compared to both mAb monotherapies as observed by better tumor growth control and median overall survival (anti-PD1 [42 day], anti-PD-L1 [50 days] and Ad5-CMV-mTNFα/mIL-2 [58 days]. However the response was lower in contrast to both combination therapies. Combining anti-PD-1 with Ad5-CMV-mTNFα/mIL-2 provided a median overall survival of 70 days (p<0.001) whereas anti-PD-L1 and Ad5-CMV-mTNFα/mIL-2 provided the greatest outcome of 78 days (p<0.001).

In poorly immunogenic (primary ICI resistant) MOC2 tumor-bearing mice (**Figure 1F**) the virus treated groups gained significantly better responses, when looking at 30 day tumor growth curves in comparison to mock (p<0.05) and anti-PD-L1 monotherapy (p<0.01). Overall survival was inferior to overall survival in MOC1-tumor-bearing mice and the only long-term surviving MOC2 tumor-bearing mouse, belonged to the experimental group anti-PD-L1 with Ad5-CMV-mTNFα/mIL-2. No long-term survivors were observed in both tumor models with mAb monotherapy, whereas combination therapy was able to control tumor growth and prolong survival over monotherapies in both tumor models. Interestingly, anti-PD-L1 monotherapy and in combination provided a better therapeutic outcome in comparison to corresponding anti-PD-1 therapy in the MOC1 tumor model. It should be noted that MOC1 growth kinetics were inhibited by high dose (10,000 VP/cell) Ad5-CMV-mTNFα but not Ad5-CMV-mIL-2 or Ad5-Luc which suggests some innate mTNFα sensitivity (**Supplementary Figure 1B**). These data show MOC tumor models exhibit ICI sensitivity and Ad5-CMV-mTNFα/mIL-2 synergizes with ICI subsequently enabling a superior anti-tumor response in murine HNSCC.

### Treatment With Non-Replicative Ad5-CMV-mTNFα/mIL-2 and Continued ICI Improves the Anti-Tumor Response in Anti-PD-1 and Anti-PD-L1 Refractory MOC1 Tumors

**Figure 2:**
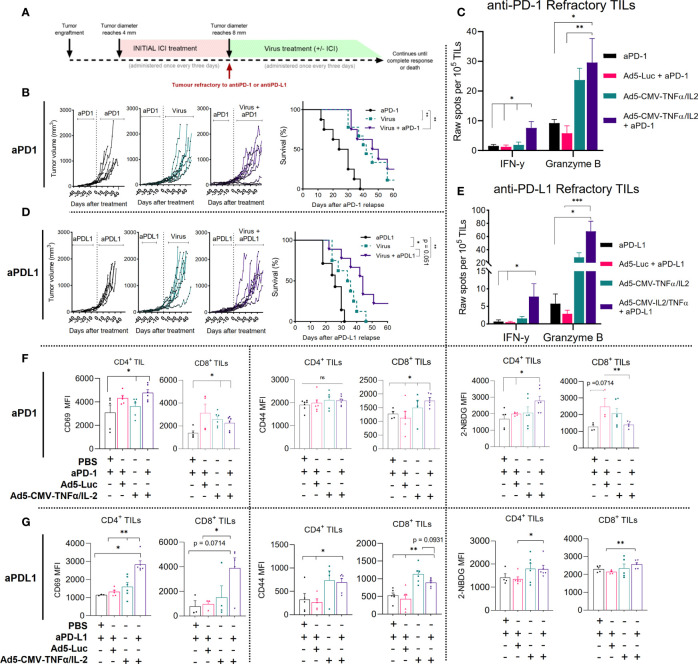
Non-replicative Ad5-CMV-mTNFα/mIL-2 improves the anti-tumor response in ICI refractory MOC1 tumors and continued ICI treatment is beneficial to the response. **(A)** Experiment design. C57BL/6J mice (n = 7-10 per group) were subcutaneously injected with MOC1 cells into the right flank. When tumors reached 4-5mm then 100ug of either anti-PD-1 or anti-PD-L1 was injected every three days intraperitoneally. When tumors progressed over 8mm, animals were assigned to a group where they were treated with either continued anti-PD-1/PD-L1, with 1 × 10^9^ VPs (non-replicative Ad5-CMV-mTNFα/mIL-2) intratumorally, or in combination. An additional virus backbone control group (Ad5-Luc) was included for immune cell analysis. Treatment frequency as indicated. **(B, D)** Individual tumor growth curves and Kaplan–Meier survival analysis for b anti-PD-1 and d anti-PD-L1 refractory. **(C, E)** Corresponding treatment group tumor derived CD3^+^ cytotoxic functional analysis measured by mGranzyme B and mIFN-y dual-ELISpot. **(F, G)** Flow cytometric analysis of T cell activation markers from MOC1 (upper panel anti-PD-1, lower panel anti-PD-L1) refractory tumors digests. *In vitro* and *ex vivo* data sets were evaluated for statistical significance by non-parametric unpaired t test and survival curves by Mantel–Cox log-rank test respectively. All data are shown as means ± SEM and significance is represented as *p < 0.05, **p < 0.01, ***p < 0.001, ns, not significant.

Next, we studied the anti-tumor response and immunological mechanisms behind Ad5-CMV-mTNFα/mIL-2 therapy in a more clinically relevant model of oral cavity cancer (**Figure 2A**). To this end, we developed an *in vivo model* of MOC1 with acquired resistance to ICI, by treatment with several rounds of either anti-PD-1 or anti-PD-L1 until animals show clear progression in spite of treatment, followed by continued ICI monotherapy, Ad5-CMV-mTNFα/mIL-2 monotherapy or the combination of both. For anti-PD-1 refractory MOC1 tumors, median overall survival for anti-PD-1 mAb monotherapy, Ad5-CMV-mTNFα/mIL-2 monotherapy and combination therapy were 27, 42 (p<0.01) and 48 days (p<0.01) respectively (**Figure 2B**). Notably, complete response was obtained with virus and anti-PD-1 in some animals. For anti-PD-L1 refractory MOC1 tumors, median overall survival for anti-PD-L1 mAb monotherapy, Ad5-CMV-mTNFα/mIL-2 monotherapy and combination therapy were 26, 35 (both p<0.05) and 44 days (p<0.01) respectively (**Figure 2D**). These data show similar median overall survival rate between the two combination groups and that virus monotherapy was more effective in extending survival of anti-PD-1 refractory mice compare to anti-PD-L1. Regardless, both combination therapy approaches maintain superior overall survival, even in an ICI refractory setting.

To analyze the functionality of TILs isolated (CD3^+^ beads) from MOC1 anti-PD-1/PD-L1 refractory mice we measured mIFN-y and mGranzyme B secretion using a dual ELISpot (**Figures 2C, E**). Overall, total mGranzyme B spot count was notably higher than mIFN-y spot count in corresponding treatment groups and spot counts was highest when combining anti-PD-1 or anti-PD-L1 with Ad5-CMV-mTNFα/mIL-2. The mIFN-y spot counts for the anti-PD-1 and anti-PD-L1 combination groups were significantly higher (p<0.05) than ICI monotherapy (fold change of 6.4 and 10.5 respectively), Ad5-CMV-mTNFα/mIL-2 monotherapy (fold change of 5.3 and 4.8 respectively), and virus backbone control (Ad5-Luc) (fold change of 8 and 14.7 respectively). Similarly, the mGranzyme B spot count for the anti-PD-1 or anti-PD-L1 combination group were significantly higher than ICI monotherapy (p<0.05) (fold change 4.37 and 11.97 respectively) and virus backbone (p<0.01) (fold change 6.1 and 26 respectively).The significant fold changes observed suggests that continued ICI treatment provides little benefit to the ICI refractory intratumoral T cell compartment and enabling cytotoxicity requires TNFα and IL-2.

We then assessed by flow cytometry activation markers associated with effector function (CD69, CD44 and 2-NDGB) expressed on tumor infiltrating CD4^+^ and CD8^+^ T cells from anti-PD-1 (**Figure 2F**) and anti-PD-L1 (**Figure 2G**) refractory mice. Combining Ad5-CMV-mTNFα/mIL-2 with either anti-PD-1 or anti-PD-L1 significantly increased (p<0.05) CD69 MFI on both CD4^+^ and CD8^+^ T cells compared to animals receiving ICI antibodies only. In addition, CD44 MFI was significantly higher (p<0.05) in all experimental groups using Ad5-CMV-mTNFα/mIL-2, in comparison to ICI monotherapy and virus backbone. Similarly, an increase in glucose uptake (a measure of metabolic fitness) quantified by 2-NBDG MFI was observed on CD4^+^ T cells in both anti-PD-1 and anti-PD-L1 experiment groups. Corresponding T infiltration and associated necrosis (HE) was further represented by an increased CD3^+^ influx of cells seen in immunohistochemistry slides from the tumor samples (stained in brown) (**Supplementary Figures S4A, B**). Altogether, these findings demonstrate that combining either anti-PD-1 or anti-PD-L1, with Ad5-CMV-mTNFα/mIL-2 provides potent stimulation and infiltration of cytotoxic T cells to the tumor microenvironment ultimately contributing to improved overall survival.

### Improvement of Activation Status and Effector Function of ICI Refractory Tumor-Infiltrating-Lymphocytes After Treatment With Non-Replicative Ad5-CMV-mTNFα/mIL-2 Corresponds to Changes in Systemic Immunity

**Figure 3:**
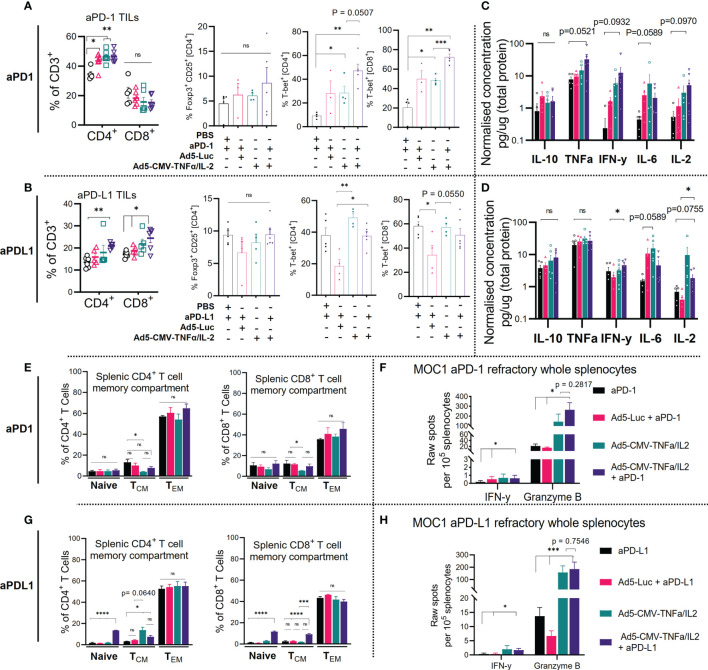
Combining ICI with non-replicative Ad5-CMV-mTNFα/mIL-2 improves trafficking and activity of ICI refractory tumor-infiltrating-lymphocytes and is reflected by changes in the secondary lymphoid organs. **(A, C)** Flow cytometric quantification of percentages of CD4^+^, CD8^+^, Foxp3^+^, T-bet^+^ expressing tumor-infiltrating lymphocytes is shown for **(A).** anti-PD-1 (upper panel) and **(C)** anti-PD-L1 (lower panel) refractory MOC1 tumor-bearing C57BL/6J mice. **(B, D)**. Bulk tumor proinflammatory cytokine analysis as measured by bead based flex set. Data are presented as logarithmic values. **(E, G)** Flow cytometric analysis of CD4^+^, CD8^+^ splenic memory compartment (naïve, effector memory [T_EM_], central memory [T_CM_]) from MOC1 anti-PD1 (upper) and anti-PD-L1 (lower) refractory mice as measured by ± expression of CD44 and CD62L. **(F, H)** Corresponding treatment group freshly resected whole splenocyte effector function analysis measured by overnight culture with mGranzyme B and mIFN-y dual-ELISpot. Flow cytometric and bulk cytokine analysis data represent two independent experiments. Statistical significance for each data set was evaluated by non-parametric unpaired t test. All data are shown as means ± SEM and significance is represented as *p < 0.05, **p < 0.01, ***p < 0.001, ****p <0.0001, ns, not significant.

We then further analyzed the tumor infiltrating lymphocyte, splenic and lymph node compartment after treatment. As expected, Ad5-CMV-mTNFα/mIL-2 treatment significantly increased the percentages of intratumoral CD4^+^ and CD8^+^ (except in anti-PD-1 refractory CD8^+^ T cells) (**Figures 3A, B**). We observed no significant changes to the percentages of immunosuppressive T reg cells (CD4^+^CD25^+^FoxP3^+^), although there was a trend (increase) in Ad5-CMV-mTNFα/mIL-2 treated groups. We also examined the expression of T-bet, the transcription factor required for type 1 immune response and mIFN-y gamma mediated T cell cytotoxicity. Notably we observed significantly more T-bet^+^ CD4^+^ and CD8^+^ cells in anti-PD-1 refractory tumors treated with ICI and Ad5-CMV-mTNFα/mIL-2 in comparison to ICI monotherapy (p<0.05). No notable differences were observed in T-bet^+^ CD8^+^ from anti-PD-L1 refractory tumors, however there was a significant increase in T-bet^+^ CD4 percentages in Ad5-CMV-mTNFα/mIL-2 treated groups compared to virus backbone (p<0.05). This finding was further implicated by bulk tumor cytokine analysis (**Figures 3C, D**) from which we identified an increased expression in all pro-inflammatory cytokines in Ad5-CMV-mTNFα/mIL-2 or combination group. Notably mIFN-y was highest in combination groups which agrees with the ELISpot data (**Figures 2C, D**) and T-bet^+^ T cells analysis when comparing to ICI monotherapy groups.

Furthermore, we profiled the peripheral lymphoid organs (inguinal lymph nodes [**Supplementary Figures S2A, B**] and spleens [**Figures 3E, G**] which revealed a significant increase to the CD4^+^ (p<0.05) and CD8^+^ (p<0.01) naïve and central memory compartment in anti-PD-L1 but not anti-PD-1 refractory mice when using combination treatment compared to mock. In contrast we observed a non-significant increase in CD4^+^ and CD8^+^ effector memory cells in anti-PD-1 refractory combination treatment group. Subsequently, we analyzed splenocytes for effector function by mIFN-y and mGranzyme B dual ELISpot (**Figures 3F, H**) which revealed a significant amount of mGranzyme B production (up to ~600 spots) in all groups treated with Ad5-CMV-mTNFα/mIL-2 and to a much lesser extent IFN-y. This reflects the results observed from the corresponding TIL data sets (**Figures 2C, D**) highlighting evidence of systemic T cell priming as a result of the treatment. Altogether these data show that Ad5-CMV-mTNFα/mIL-2 enables a cytotoxic T cell program which partially involves T-bet mediated type 1 T cell induction. Given the dominant expression of mGranzyme B over IFN-y, it is likely that other functional T cell lineages that require alternative transcriptional regulators are involved.

### Treatment of Anti-PD-L1 Refractory MOC1 Tumors With Non-Replicative Ad5-CMV-mTNFα/mIL-2 Improves the Metabolic Profile of Tumor-Infiltrating-Lymphocytes

**Figure 4:**
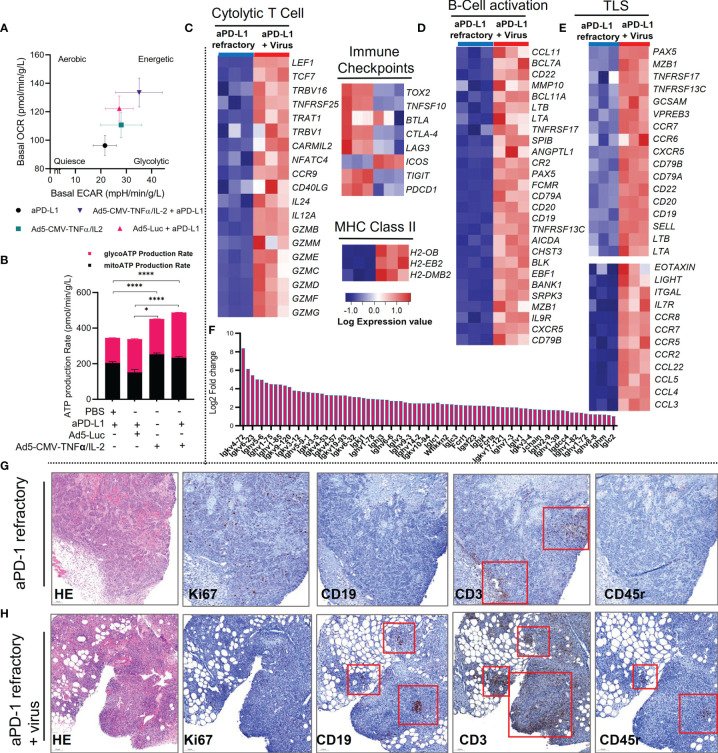
Combining anti-PD-L1 with non-replicative Ad5-CMV-mTNFα/mIL-2 improves tumor-infiltrating T cell bioenergetics and addition of virus induces a tertiary lymphoid structure gene signature in MOC1 ICI refractory tumors. **(A)** Bioenergetics map of MOC1 anti-PD-L1 refractory tumor-infiltrating lymphocytes (CD3^+^) isolated by magnetic bead (negative selection) approach and analyzed by Seahorse XF analyzer. Data is presented by basal oxygen consumption rate (OCR) and extracellular acidification rate (ECAR). **(B)** Corresponding real-time ATP production rate by glycolysis and aerobic respiration as quantified by Seahorse XF analyzer software. **(C–E)** Bulk RNA-Seq of CD45^+/-^ populations isolated by from MOC1 anti-PD-L1 refractory tumors by magnetic bead approach. Heat maps for significantly differentially expressed genes between anti-PD-L1 refractory tumors and anti-PD-L1 refractory tumors treated with Ad5-CMV-mTNFα/mIL-2. Heat maps are organized into **(C)** T cell activation genes, immune checkpoints and MHC genes **(D)** B-cell activation genes, **(E)** typical tertiary lymphoid structure genes. **(F)** Fold change increase of significantly differentially expressed genes related to immunoglobulin synthesis between anti-PD-L1 refractory tumors and anti-PD-L1 refractory tumors treated with Ad5-CMV-mTNFα/mIL-2. **(G, H)** Immunohistochemistry of tertiary lymphoid structure related markers (HE, Ki67, CD19, CD3 and CD45r) from anti-PD-1 refractory tumors treated with **(H)** or without **(G)** non-replicative Ad5-CMV-mTNFα/mIL-2. Red boxes highlight positively stained areas (brown) for respective marker indicated in the bottom left of each panel. DESeq2 was used to compare gene expression between groups and the Walkd test was used to generate p-values and log2 fold changes. Genes with an adjusted p-value < 0.05 and absolute log2 fold change > 1 were determined as differentially expressed genes. Statistical significance for ATP production rate was evaluated using one-way ANOVA and presented as means ± SEM with significance represented as *p < 0.05, and ****p < 0.0001.

Next, we investigated the metabolic and transcriptomic changes to the tumor immune microenvironment of anti-PD-L1 refractory MOC1 tumors upon treatment with Ad5-CMV-mTNFα/mIL-2. Analysis of CD3^+^ T cells from virus-treated tumors revealed an overall increase in bioenergetics (aerobic glycolysis) as measured by basal oxygen consumption rate (OCR) (pmol/min/g/L) (p=<0.05 when comparing combination therapy to ICI monotherapy) and basal extracellular acidification rate (ECAR) (mpH/min/g/L) (p=<0.01 and p=< 0.05 when comparing combination therapy to ICI monotherapy and Ad5-Luc therapy, respectively)(**Figure 4A** and **Supplementary Figure S1C**). Unexpectedly, Ad5-Luc + anti-PD-L1 induced a higher OCR rate than Ad5-CMV-mTNFα/mIL-2 monotherapy. This could be caused by infiltration of antiviral memory T cells against adenovirus backbone. Furthermore, continued administration of anti-PD-L1 with virus provided the most metabolically active profile out of all treatment groups. This finding was further confirmed (**Figure 4B**) by quantification of overall CD3^+^ T cell ATP production rate which showed a significant increase in glycolysis derived ATP production in the virus monotherapy (450.6 pmol/min/g/L) and to further extent in the combination group (487.3pmol/min/g/L) compared to ICI monotherapy and Ad5-Luc monotherapeutic groups.

Consistent with the metabolic and functional profiling of antiPD-L1 refractory MOC1-derived CD3^+^ T cells, differential gene expression analysis, revealed a strong upregulation of a cytolytic and cytotoxic gene signature (*GZMB, GZMM, GZME, GZMC, GZMD, GZMF, GZMG, IL-24, IL-12A and CD40LG*) following treatment of anti-PD-L1 mAb refractory tumors with Ad5-CMV-mTNFα/mIL-2 (**Figure 4C**). This signature was accompanied by downregulation of genes associated with immune checkpoints (*CTLA-4, LAG3, ICOS, TIGIT, and PCDC1*), upregulation of MHC Class II genes (*H2-OB, H2-EB2, and H2-DMB2*) and memory-like characteristics (*TCF7 and IL7R*). These findings strongly support the functional analysis of the intratumoral and systemic T cell compartment.

### Treatment of Anti-PD-L1 Refractory MOC1 Tumors With Non-Replicative Ad5-CMV-mTNFα/mIL-2 Induces a Tertiary Lymphoid Structure Gene Signature

Further examination of the tumor immune compartment transcriptome, suggest an even stronger, activated B-cell profile (**Figure 4D**). This finding led us to identify upregulation of several tertiary lymphoid structure (TLS) hallmark associated genes (*CXCR5, CD79B, CD19, CD20, SELL, LTa, LTb, LIGHT and CCR7*) (**Figure 4E**) including upregulation of genes important for germinal-center initiation (*CR2, POU2AF1, EBF1, SPIB, BACH2*). To confirm these data, we examined histologic analysis of tumor Ki67^+^, CD19^+^, CD3^+^, CD45r^+^ cells and identified evidence of TLS-like clusters in virus treated groups (**Figure 4H**) compared to ICI monotherapy (**Figure 4G**) where only CD3^+^ clusters were identified. We also observed upregulation of multiple genes related to somatic hyper mutation and class switching (*IGKV4-72, IGHV5-6, IGLV3, IGHM, FCRL1, FCRLA* and *JCHAIN*) (**Figure 4F**). We validated these findings by observing an increase in CD19^+^% in both anti-PD-L1 and anti-PD-1 refractory MOC1 tumors treated with Ad5-CMV-mTNFα/mIL-2 (**Supplementary Figure S3A**). This finding and our observation of improved responses to ICI is in agreement with recent reports that suggest TLS promote response to immunotherapy (28, 29)

### Combining ICI With Non-Replicative Ad5-CMV-mTNFα/mIL-2 Improves Tertiary Lymphoid Structure Associated Antitumor Response in MOC1 Tumors

**Figure 5:**
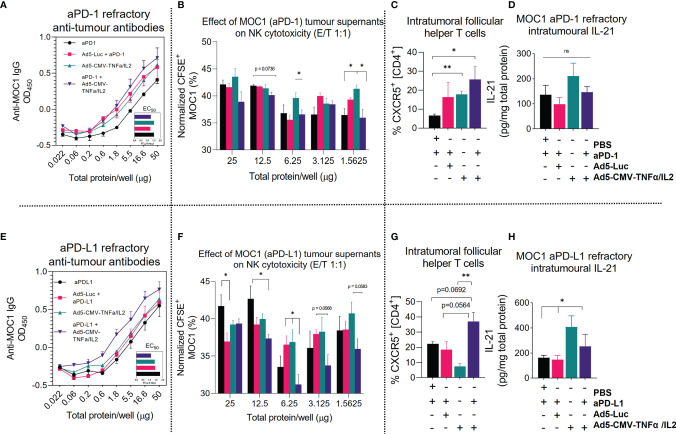
Combining ICI with non-replicative Ad5-CMV-mTNFα/mIL-2 improves tertiary lymphoid structure associated antitumor response. **(A, E)** Anti-MOC1 antibodies derived from MOC1 anti-PD-1/L1 refractory tumor digests supernatants and their respective treatment groups cultured with MOC1 cell line and measured by total IgG^+^ signal using In-cell ELISA. **(B, F)** corresponding effects of whole tumor digests on NK-mediated cell killing of MOC1 *in vitro*. NK cells were derived from fresh C57BL/6J mouse spleens and isolated by negative selection magnetic bead based approach. **(C, G)** Flow cytometric analysis of CD4^+^, CD8^+^, CXCR5^+^, tumor infiltrating lymphocytes from **(C)** anti-PD-1 and **(G)** anti-PD-L1 refractory mice. **(D, H)** Measurement of intratumoural IL-21 concentrations as measured by ELISA and normalized to total protein concentration. Data sets were evaluated for statistical significance by non-parametric unpaired t test and shown as means ± SEM with significance represented as *p < 0.05, **p < 0.01, ns, not significant.

To offer functional insight and having identified upregulation of several genes involved in immunoglobulin synthesis (**Figure 4F**), we evaluated the presence of anti-tumor associated antibodies and NK cell stimulating activity using supernatants extracted treated tumors and isolated NK cells from mouse spleens. Indeed, compared to ICI monotherapy, we identified higher anti-MOC1 associated IgG titers after armed and unarmed virus treatment (in both anti-PD-1 and anti-PD-L1 refractory tumors), but the highest anti-tumor antibody values identified were in the combination treatment group (**Figures 5A, E**). The most notable positive increase in anti-tumor antibody production was identified in the anti-PD-L1 combination group, even when comparing to the corresponding anti-PD-1 treatment group. This was consistent with the findings observed when analyzing the effects of the tumor supernatant on NK-mediated cytotoxicity. From this data set we identified that the antiPD-L1 with virus combination group enabled superior NK-mediated MOC1 cell killing compared to all other experimental groups (**Figure 5F**). This was less notable when testing anti-PD-1 refractory MOC1 tumor supernatants (**Figure 5B**). To complement these findings, we analyzed intratumoral T cells expressing the lymphoid follicle homing chemokine receptor CXCR5 (follicular B helper T cells) and IL-21, critical markers of germinal center formation, NK maturation and also formation of follicular B helper T cells. We found an increase in CD4^+^ CXCR5^+^ T cells in both anti-PD-1 and anti-PD-L1 combination treated groups in comparison to ICI monotherapy and virus backbone (**Figures 5C, G**). Furthermore, we observed higher IL-21 production in tumors treated with Ad5-CMV-mTNFα/mIL-2, which was more notable in the anti-PD-L1 refractory treatment groups (p<0.05) (**Figures 5D, H**). Altogether these data show that Ad5-CMV-mTNFα/mIL-2 enables germinal center formation and production of anti-tumor antibodies which have the potential to enable NK-mediated anti-tumor response. Interestingly, this is more evident in anti-PD-L1 refractory tumors suggesting a distinctive role of anti-PD-L1 in regulating the response to virus therapy.

## Discussion

In this study, we demonstrate that combinatorial treatment with anti-PD-1 or anti-PD-L1 blocking mAb and non-replicative Ad5-CMV-mTNFα/mIL-2 improve**s** the anti-tumor activity of the intratumoral immune compartment in the context of HNSCC. The use of virus treatment represents a benefit for ICI refractory HNSCC tumors, because the combination strategy enhances both the adaptive cell immune response, through stimulation of cytotoxic T cells, and the humoral response, through generation of anti-tumor antibodies. This was associated with the formation of TLS in virus treated tumors. This has important implications, given the prognostic value associated with pre-existing TLS on progression-free survival, and an ongoing effort on developing strategies for enhancing the intratumoral B cell response in HNSCC patients (30–32). We also show our oncolytic adenovirus (Ad5/3-E2F-D24-hTNFa-IRES-hIL-2) has lytic cancer cell killing capacity and robust transgene expression in multiple human HNSCC cell lines. Collectively, these findings indicate that our approach might be a useful treatment option for HNSCC patients.

A key finding of this study is the ability of the mTNFα and mIL-2 encoding adenovirus to initiate a distinctive TLS-gene signature when combined with anti-PD-L1 mAb therapy in an anti-PD-L1 refractory model. This novel mechanistic insight might explain the observed synergy between the virus and ICI, as supported by recent reports describing the benefit of preexisting tumor associated TLS to the response of immunotherapy. The mechanism of how TLSs favorably affect the responses are not fully understood and require further investigation. However, reports suggest TLSs have a key role in sustaining an immune-responsive microenvironment (28, 29). Performing multi-color immunofluorescence immunohistochemistry in future studies is needed to confirm our findings. It may be the case that the mTNFα and mIL-2 coding adenovirus virus induces transient (tumor regression) non-classical TLS (33). Additionally, it remains to be confirmed how mTNFα, mIL-2 and the generation of acquired ICI resistance (through anti-PD-L1 treatment) contributes to the TLS signature, perhaps the latter providing a tumor priming effect. Indeed, previous reports have described increased TLS formation following ICI monotherapy. Likewise, although not representative of all ICI resistant phenotypes, we have previously reported a phenotypic change to the tumor microenvironment transcriptome following generation of anti-PD-1 resistance in a B16.OVA melanoma mouse model (21, 34, 35). These changes indicated suppression of T cell related genes. Also, although we have previously studied effects of adenovirus derived mTNFα and mIL-2 in tumor immunomodulation, further investigation is required to fully delineate their individual roles in the context of TLS formation (36). It should be mentioned that improved tumor growth control and a similar increase in percentage of CD19^+^ as well as NK1.1^+^ cells were identified when combining mTNFα and mIL-2. Generally, mIL-2 appeared to have a more significant impact on immunomodulation than mTNFα, however additional function studies are required to support these findings (17). Indeed, other studies have described the role of IL-2 in B cell activation and differentiation to antibody producing plasma cells (37). Moreover, given the critical role of local antigen stimulation, TNFα associated type I receptor signaling as well as LTa/LTβ associated type 3 receptor signaling in TLS priming and maturation (all of which are directly and indirectly provided by our virus), it would not be unexpected to observe TLS formation following virus treatment (38–42).

Understanding how continued ICI treatment regulates the extent to which TLS are either immunosuppressive or anti-tumorigenic in the context of our study remains to be confirmed in further studies. It is known that in many human cancers PD-1 and its ligands are expressed on several types of tumor infiltrating immune cells, including TLS-T and TLS-B cells (43, 44). The major TLS-T cell types, which include T-bet^+^ T helper 1 cells (T_H_1), PD-1^+^ T follicular helper cells (T_FH_) and Tregs are regulated on the PD-1 axis prior and during lineage commitment, ultimately determining the immune modulating nature of the cytokines produced (44–47). This notion of a cytotoxic TLS, observed in our study when combining anti-PD-L1 with virus, is supported by a series of studies which revealed adenoviruses innately prime CD4^+^ T cells with cytotoxic potential (with a distinct GZMB^+^ signature corresponding to the observations from our study), and that this unique signature is regulated by PD-1, LAG3 and the availability of IL-2 (15, 48). These regulatory signals that are directly provided by our combinatorial treatment approach subsequently highlight the critical interplay between this virus with ICI (16).

The improved therapeutic outcome observed in our combination therapy approach is unlikely restricted to the proposed generation and maintenance of cytotoxic CD4^+^ T cells because a strong activation signature on the CD8^+^ T cell compartment was also identified. Tumor infiltrating activated cytotoxic T cells are likely composed of T cells recognizing adenovirus antigens, and rejuvenated tumor antigen-specific T cells. This is consistent with our understanding of IL-2 and ICI in circumventing T cell exhaustion and TLSs which are recognized as a place where T cell and B cell responses occur (28, 29). Others have demonstrated this using models of exhausted CD8^+^ T cells during chronic lymphocytic choriomeningitis mammarenavirus infection and their subsequent functional restoration following ICI treatment (49). We have also previously shown significant renewal of OVA/gp100/Trp2 specific CD8^+^ T cells in the peripheral lymphoid tissues of B16-OVA tumor-bearing mice, which was again most evident in the ICI plus virus treatment group (21). With that said, it would be worth investigating this combinatorial treatment approach in the context of HPV^+^ HNSCC tumors, given that persistent HPV infection (mediated by exhausted CD8^+^ T cells) is associated with increased risk for disease recurrence following surgical resection (50–54).

The benefit of using PD-1 and PD-L1 blocking mAbs with this virus is now clearly not limited to positively regulating T cell function. PD-1^+^ regulatory B cells (Bregs) can suppress anti-tumor immunity through suppression of cytotoxic T cell function, a process regulated *via* TNFα signaling and PD-1 expression (55, 56). In addition, PD-1 has been shown to regulate PD-L1/PD-L2^+^ germinal center B cell survival and the formation and affinity of long-lived plasma cells through influencing signals that control the rate of germinal center cell death (57). Indeed, this could explain the increase in tumor-specific antibodies observed in the anti-PD-L1 combination group, which were also indirectly shown to enhance NK-mediated ADCC and which might facilitate antibody-dependent cellular phagocytosis (ADCP), complement-dependent cytotoxicity (CDC) or increase antigen presentation by antigen-presenting cells such as macrophages and dendritic cells (58). In future studies, it would be worth further phenotyping the B-cell and NK-cell response in conjunction with multi-cell depletion studies to shed light on this immune compartment and its contribution to the overall anti-tumor response.

In summary, adenoviruses armed with IL-2 and TNFα are able to significantly improve therapeutic outcome in ICI sensitive, primary resistant and acquired resistant models of HNSCC, likely through immunogenic viral oncolysis and induction of a cytolytic immune response associated with tertiary lymphoid structure formation. Moreover, in all three models, we observed a synergistic effect when combining virus with anti-PD-1 or anti-PD-L1, evident at a mechanistic level and by improvement to overall survival. These data provide the rationale for a clinical trial, where Ad5/3-E2F-D24-hTNFa-IRES-hIL-2 would be administered to ICI refractory or resistant HNSCC patients, together with an ICI, in order to optimize clinical responses.

## Data Availability Statement

The datasets presented in this study can be found in online repositories. The names of the repository/repositories and accession number(s) can be found below: https://www.ebi.ac.uk/arrayexpress/E-MTAB-11204.

## Ethics Statement

The animal study was reviewed and approved by National Animal Experiment Board (Eläinkoelautakunta ELLA) and the Provincial Government of Southern Finland (license number ESAVI/28404/2019).

## Author Contributions

JC, JS, VCC, DQ, TS, VL, ST, and AH designed the experiments. JC, TK, CH, SB, SP, and VZ conducted the experiments. JC, TK, RH, SS, SP, KA, and LB analyzed the results. All the authors contributed to writing and reviewing the manuscript. All authors contributed to the article and approved the submitted version.

## Funding

This study received funding from the European Union’s Horizon 2020 research and innovation programme under the Marie Skłodowska-Curie grant agreement No 813453. This study was supported by Jane and Aatos Erkko Foundation, HUCH Research Funds (VTR), Finnish Cancer Organizations, University of Helsinki, Novo Nordisk Foundation, Päivikki and Sakari Sohlberg Foundation, TILT Biotherapeutics Ltd. We thank Albert Ehrnrooth and Karl Fazer for research support. The funders were not involved in the study design, collection, analysis, interpretation of data, the writing of this article or the decision to submit it for publication.

## Conflict of Interest

Authors JC, VCC, JMS, RH, SS, AH were employed by the company TILT Biotherapeutics Ltd.

The remaining authors declare that the research was conducted in the absence of any commercial or financial relationships that could be construed as a potential conflict of interest.

The authors declare that this study received funding from TILT Biotherapeutics Ltd. The funder had the following involvement in the study: permission to use oncolytic adenovirus TILT-123.

## Publisher’s Note

All claims expressed in this article are solely those of the authors and do not necessarily represent those of their affiliated organizations, or those of the publisher, the editors and the reviewers. Any product that may be evaluated in this article, or claim that may be made by its manufacturer, is not guaranteed or endorsed by the publisher.

## Supplementary Material

Supplementary Figure S1A
**(A)** Lytic activity of Ad5/3-E2F-D24 in HNSCC patient derived cell lines. Cells were cultured over 10 days with either 10, 100 or 1000 VP/cell and viability was measured by MTS. Data represents 3 independent experiments and all data are shown as means ± SEM. *In vitro* data sets were evaluated for statistical significance by non-parametric unpaired t tests Statistical significance is represented as *p<0.05, **p<0.01, ***p<0.001, and ****p<0.0001.

Supplementary Figure S1B Growth kinetics of murine oral cancer cell line (MOC1). Cells were seeded onto xCELLigence 16 well plates and after 24 hours were infected with either Ad5-CMV-mIL-2, Ad5-CMV-mTNFα, Ad5-CMV-mTNFα/mIL-2 or Ad5-Luc at 10, 100, 1000 and 10000 VP/cell. Impedance was measured by the real-time xCELLigence cell analyzer for 100 hours. Data was normalized to 24 hour time point and is presented as mean cell index from triplicates.

Supplementary Figure S1C Basal OCR and ECAR of MOC1 anti-PD-L1 refractory CD3^+^ as shown and described in Figure 4A. Data represents 2 independent experiments, carried out in >6 technical replicates and all data are shown as means ± SEM. Statistical significance were evaluated for statistical significance by non-parametric unpaired t tests with significance represented as *p<0.05 and **p<0.01.

Supplementary Figure S1D Average lytic activity measured by MTS of oncolytic adenoviruses (Ad5/3-E2F-D24-hTNFa-IRES-hIL-2 and Ad5/3-E2F-D24) in all HNSCC patient derived cell lines cultured over 10 days with either 10, 100 or 1000VP/cell. Data are shown as means ± SEM. Statistical significance were evaluated by two-way ANOVA with Tukeys post test with significance represented as *p<0.05, **p<0.01, ***p<0.001, and ****p<0.0001.

Supplementary Figure S2A, B Flow cytometric analysis of anti-PD-1 and anti-PD-L1 refractory lymph node T cell memory compartment. Lymph node digests from MOC1 ICI refractory mice were digested by mechanical disruption and were stained with fluorescent conjugated antibodies against CD3, CD4, CD8, CD44 and CD62L and are presented as naïve (CD44^-^, CD62L^+^), effector memory [TEM] (CD44^+^, CD62L^-^) or central memory [TCM] (CD44^+^, CD62L^+^). Data sets were evaluated for statistical significance by non-parametric unpaired t test and shown as means ± SEM with significance represented as *p<0.05, **p<0.01, ***p<0.001.

Supplementary Figure S3A Quantification of CD19^+^ cells in MOC1 anti-PD-1 and anti-PD-L1 refractory treated tumors from IHC. Graphical representation of %CD19^+^ cells in MOC1 ICI refractory tumors. Data sets were evaluated for statistical significance using one-way ANOVA and shown as means ± SEM with significance represented as *p<0.05, **p<0.01.

Supplementary Figure S3B, C Correlation analysis of TILT-123 E1a (D24) expression vs IL-2/TNFα secretion following infection of HNSCC patient derived cell lines. Spearman non-parametric correlation analysis between E1a expression and IL-2 3b production and 3c TNFα production from TILT-123 infected HNSCC cell lines.

Supplementary Figure S4A, B Immunohistochemistry of tumors from respective treatment groups from MOC1 anti-PD-1/PD-L1 refractory animal experiment. The treatment groups included ICI monotherapy, Ad5-Luc + ICI control, Ad5-CMV-mTNFα/mIL-2 monotherapy and Ad5-CMV-mTNFα/mIL-2 + ICI combination therapy. Tumors from respective groups were fixed, paraffin embedded and stained for H&E and CD3 (brown).

## Abbreviations

ADCC, antibody-dependent cell-mediated cytotoxicity; ADCP, antibody-dependent cellular phagocytosis; CDC, complement-dependent cytotoxicity; DMEM, Dulbecco’s Modified Eagle Medium; ECAR, extracellular acidification rate; HE, haematoxylin and eosin; HNSCC, head and neck squamous cell carcinoma; HPV, human papilloma virus; ICI, immune checkpoint inhibitor; IL-2, interleukin 2; mIFN-y, interferon gamma; NK, natural killer; OCR, oxygen consumption rate; PFA, paraformaldehyde; PD, programmed death; SEM, standard error of the mean; TFH, T follicular helper cell; TH1, T helper 1 cell; TLS, tertiary lymphoid structure; TNFα, tumor necrosis factor alpha; Treg, T regulatory cell.
