# Supplementary figures and images for: Adenovirus Encoding Tumor Necrosis Factor Alpha and Interleukin 2 Induces a Tertiary Lymphoid Structure Signature in Immune Checkpoint Inhibitor Refractory Head and Neck Cancer
Source: Front Immunol. 2022 Mar 7;13:794251. doi: 10.3389/fimmu.2022.794251 (PMC8959099; doi:10.3389/fimmu.2022.794251)

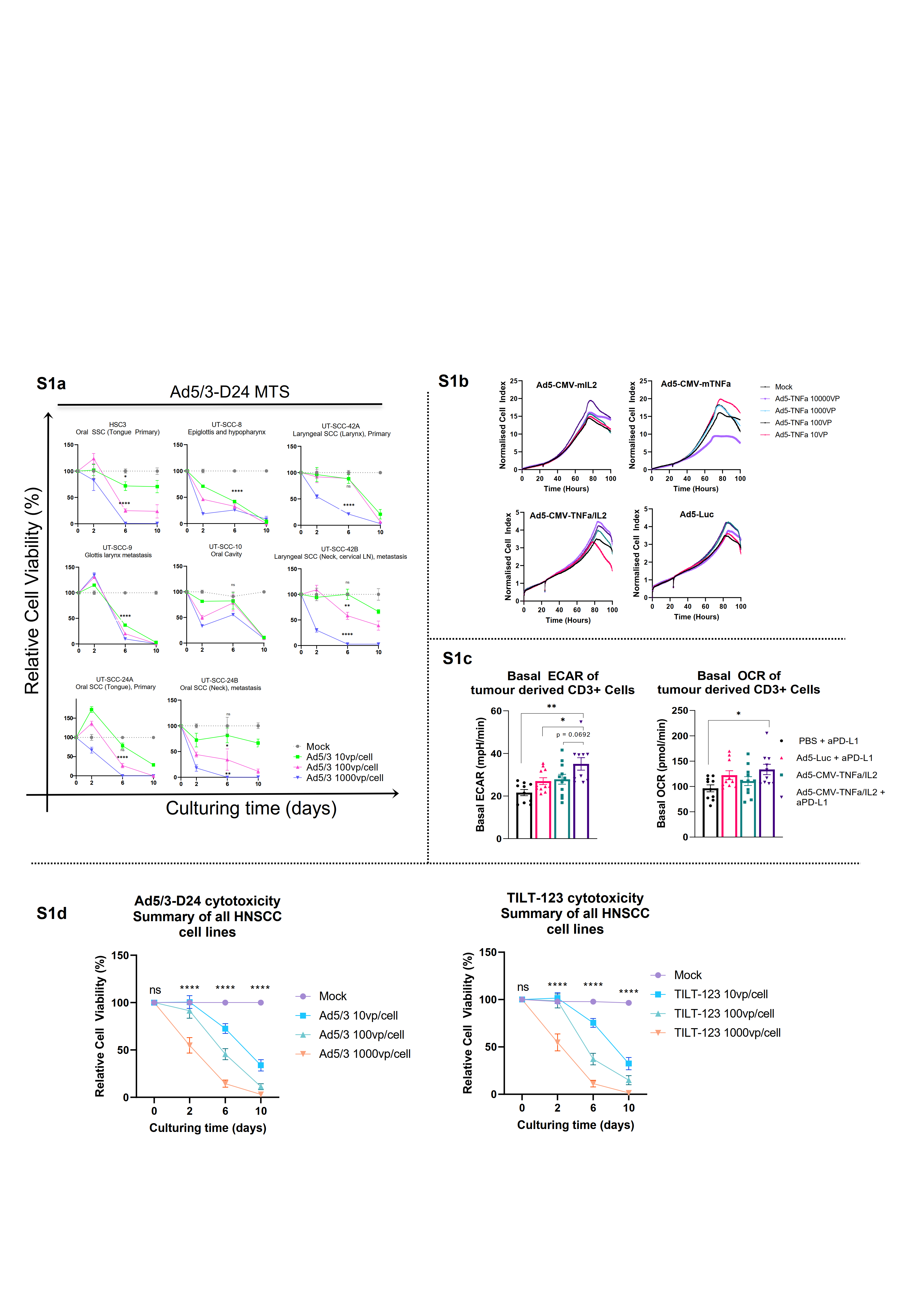

Supplement: Supplementary Figure S1A — (A) Lytic activity of Ad5/3-E2F-D24 in HNSCC patient derived cell lines. Cells were cultured over 10 days with either 10, 100 or 1000 VP/cell and viability was measured by MTS. Data represents 3 independent experiments and all data are shown as means ± SEM. In vitro data sets were evaluated for statistical significance by non-parametric unpaired t tests Statistical significance is represented as *p<0.05, **p<0.01, ***p<0.001, and ****p<0.0001. [file Image_1.tif]

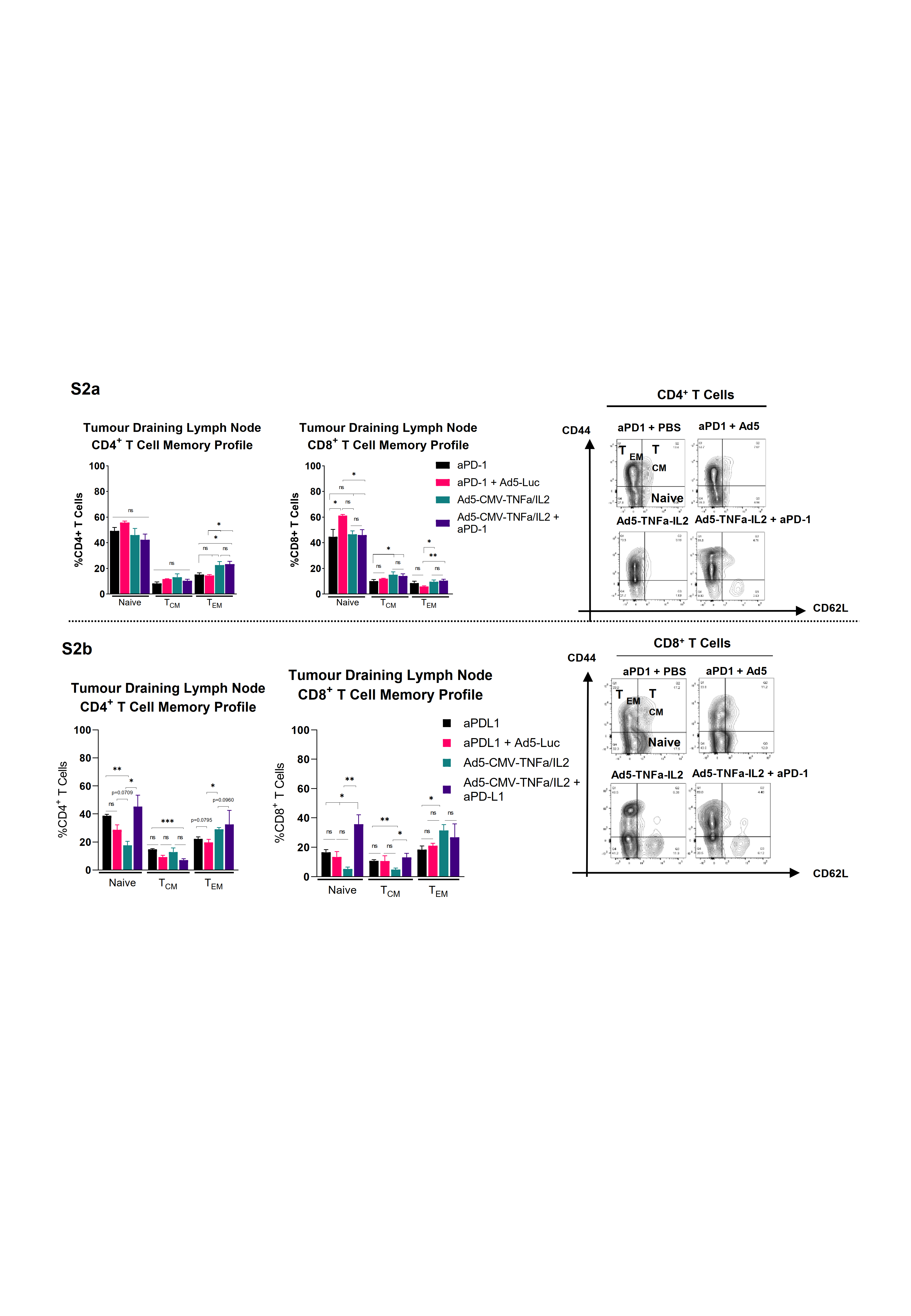

Supplement: Supplementary Figure S2A, B — Flow cytometric analysis of anti-PD-1 and anti-PD-L1 refractory lymph node T cell memory compartment. Lymph node digests from MOC1 ICI refractory mice were digested by mechanical disruption and were stained with fluorescent conjugated antibodies against CD3, CD4, CD8, CD44 and CD62L and are presented as naïve (CD44-, CD62L+), effector memory [TEM] (CD44+, CD62L-) or central memory [TCM] (CD44+, CD62L+). Data sets were evaluated for statistical significance by non-parametric unpaired t test and shown as means ± SEM with significance represented as *p<0.05, **p<0.01, ***p<0.001. [file Image_2.tif]

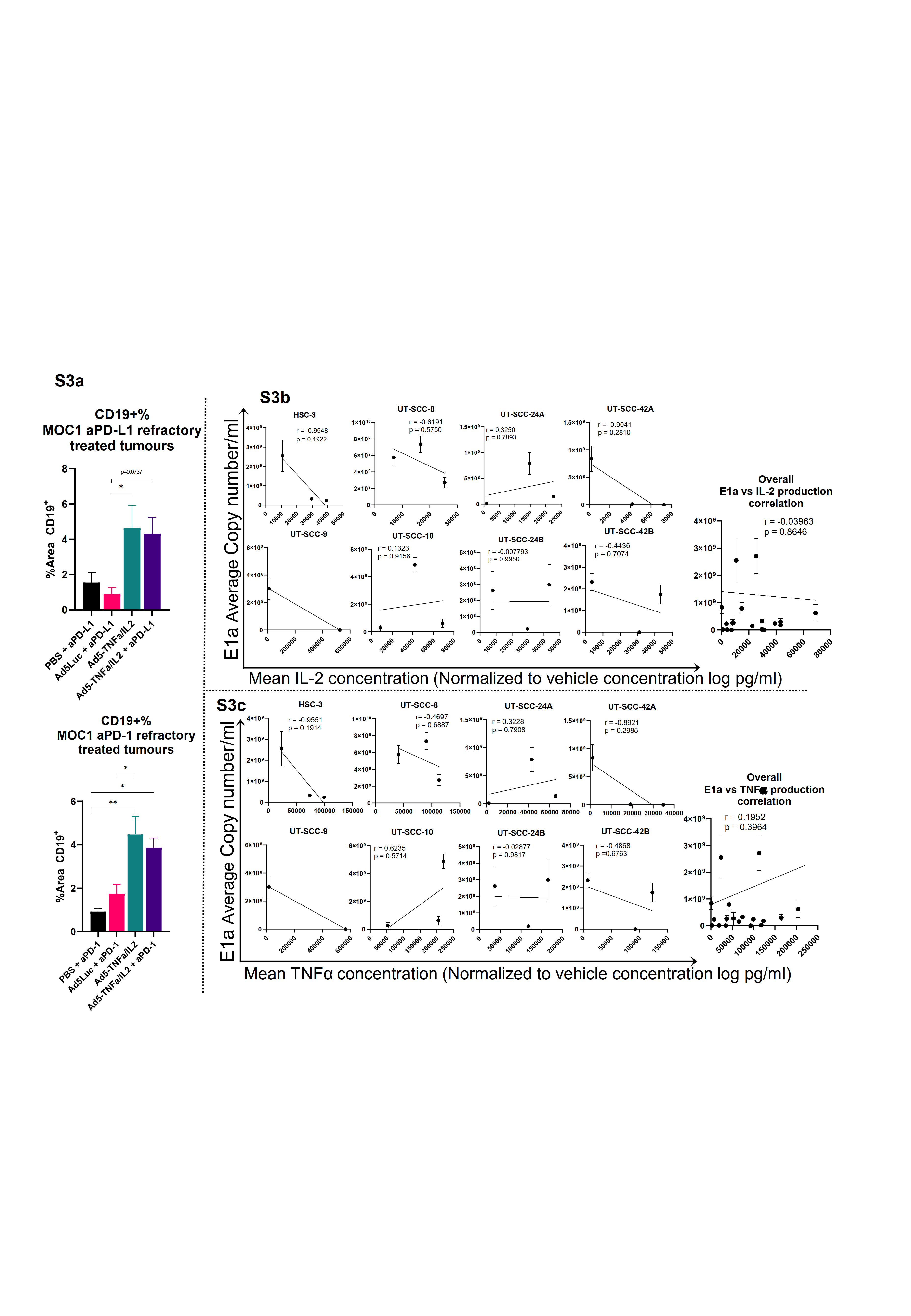

Supplement: Supplementary Figure S3A — Quantification of CD19+ cells in MOC1 anti-PD-1 and anti-PD-L1 refractory treated tumors from IHC. Graphical representation of %CD19+ cells in MOC1 ICI refractory tumors. Data sets were evaluated for statistical significance using one-way ANOVA and shown as means ± SEM with significance represented as *p<0.05, **p<0.01. [file Image_3.tif]

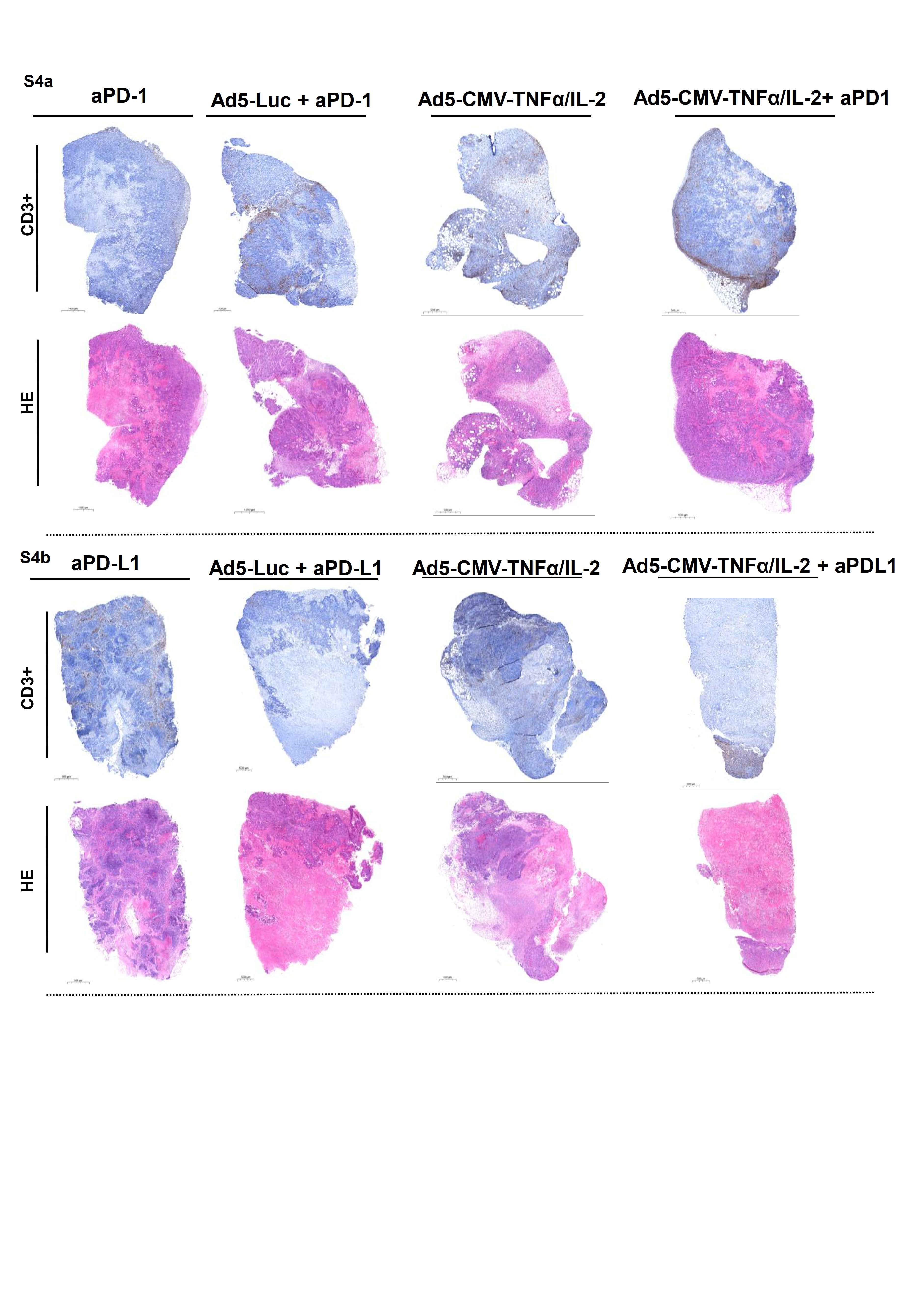

Supplement: Supplementary Figure S4A, B — Immunohistochemistry of tumors from respective treatment groups from MOC1 anti-PD-1/PD-L1 refractory animal experiment. The treatment groups included ICI monotherapy, Ad5-Luc + ICI control, Ad5-CMV-mTNFα/mIL-2 monotherapy and Ad5-CMV-mTNFα/mIL-2 + ICI combination therapy. Tumors from respective groups were fixed, paraffin embedded and stained for H&E and CD3 (brown). [file Image_4.tif]
